# Measuring Colloidal Forces With Atomic Force Microscopy 1: Salt Influence on Hydrophobic and Hydrophilic Interactions

**DOI:** 10.1002/jemt.24832

**Published:** 2025-02-22

**Authors:** Luis N. Ponce‐Gonzalez, Wisnu Arfian A. Sudjarwo, José L. Toca‐Herrera

**Affiliations:** ^1^ Institut für Biophysik, Department für Bionanowissenschaften Universität für Bodenkultur Wien Vienna Austria; ^2^ Research Center for Polymer Technology, National Research and Innovation Agency, Republic of Indonesia (BRIN) Tangerang Selatan Indonesia

**Keywords:** atomic force microscopy, colloid stability, contact angle, DLVO, fluorocarbon, hydrophilic, hydrophobic, polystyrene, surface interactions, zeta potential

## Abstract

Colloidal forces are essential for maintaining the stability and functionality of colloidal systems, affecting various industrial, biological, and environmental processes. They play an important role in determining the behavior of particles in suspensions, including stability, aggregation, and surface interactions. In this primer, we present basic concepts and protocols for studying colloidal interactions at different salt concentrations using atomic force microscopy (AFM). Following this methodology, hydrophilic substrates (i.e., silica) were easily functionalized with a hydrophobic fluorocarbon (1H,1H,2H,2H‐Perfluorooctyltrimethoxysilane, FOTS) via chemical vapor deposition (CVD) and characterized by the sessile drop method, electrophoretic light scattering, AFM imaging, and scanning electron microscopy (SEM) to determine parameters such as contact angle, zeta potential, and surface roughness, respectively. Thus, after the preparation and characterization of a well‐defined colloidal system, force‐distance experiments using AFM allowed for the measurement of hydrophobic and hydrophilic interactions in salt solutions. Furthermore, we describe in detail the processing and fitting of the experimental data with an extended DLVO model.

## Graphical abstract

Colloidal forces play an important role in many life phenomena. Here, we present the preparation of hydrophobic and hydrophilic systems for the study of colloidal forces by AFM. The data are modeled using an extended DLVO model.

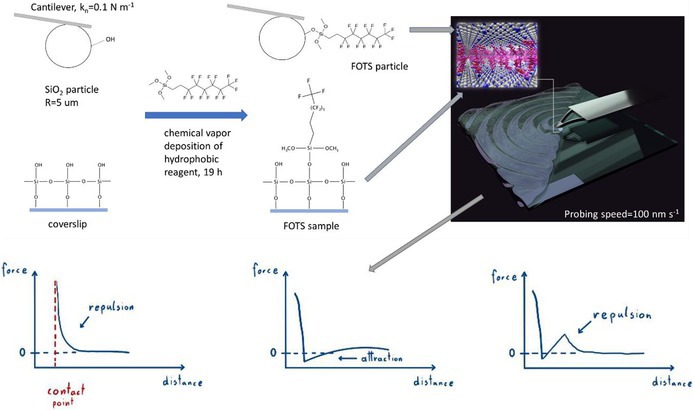

**Summary**

- Atomic force microscopy (AFM) can be used to investigate chemical surface modification.
- AFM allows direct measurement of surface forces in hydrophobic and hydrophilic colloidal systems.
- An extended DLVO model, including hydration and hydrophobic interactions, is used to fit the experimental data.

## 1 Introduction

Colloidal forces govern the behavior of particles in a solvent, influencing stability, aggregation, and surface interactions (Belloni 2000; Liang et al. 2007). Examples of colloidal systems include blood, biofilms, milk, wine, paints and coatings, and cosmetics (Ogemdi 2019). Consequently, colloidal interactions are crucial for the interpretation of many phenomena in physics, chemistry, and biology.

Among the interactions that occur in colloidal systems, hydrophobic and hydration forces remain poorly understood. Hydrophilic substrates such as silica are widely used as support in composites with grafted organic molecules for applications in catalysis and biomedicine (Wei et al. 2022; Huang et al. 2022). Therefore, it is of interest to study how organic layers covering hydrophilic substrates influence their overall colloidal interactions. For example, some studies have examined the influence of ionic valency on the interactions of silica‐supported organic chains with terminal amino and thiol groups (Liu et al. 2017). Furthermore, hydrophobic colloidal forces can help define the interactions involved in biochemical processes, including receptor‐ligand binding and molecular assembly (Sun 2022). The development of techniques such as surface force apparatus (SFA) and atomic force microscopy (AFM) has enabled the direct measurement of the forces involved in the aforementioned interactions under different solvent and temperature conditions (Israelachvili and Adams 1976; Toca‐Herrera 2019; Müller et al. 2021).

The DLVO theory, developed by Derjaguin–Landau–Verwey–Overbeek, has been extensively used to model colloidal interactions in salt solutions, including electric double‐layer (EDL) and van der Waals (vdW) forces (Derjaguin and Landau 1941; Verwey 1947; Israelachvili 2011). However, this theory alone cannot explain the diversity of experimental behaviors observed in different hydrophilic and hydrophobic systems (Tabor et al. 2014). To address this, extended expressions of DLVO theory have been developed, incorporating models for hydrophilic repulsion (i.e., hydration force) and hydrophobic attraction as exponential decay functions (Donaldson Jr. et al. 2015). However, despite considerable experimental and theoretical efforts, the intrinsic origin and scope of the hydrophobic force remain unclear. Proposed mechanisms include polar molecule rearrangement at hydrophobic interfaces (entropic effects), local charge fluctuations (electrostatic effects), micro‐ and nanobubble bridging, and vapor cavitation (Wang et al. 2023).

In the present work, we describe a simple method to study colloidal interactions in hydrophobic and hydrophilic systems at varying salt concentrations. While essentially a primer, we report new findings concerning how fluorocarbon hydrophobization affects the colloidal interactions of hydrophilic substrates. Our aim is to provide a detailed protocol for substrate functionalization, characterization, and force‐distance measurements using AFM. In addition, we outline the steps for processing and analyzing AFM data and fitting it to an extended DLVO model.

## 2 Materials and Methods

### 2.1 AFM Measurements

Atomic force microscopy (AFM) force‐distance measurements were performed with a Nanowizard 1 AFM (Bruker, Germany) using contact mode at 295.15 K. The cantilevers used were tipless silicon nitride MLCT‐O10 (Bruker) with a nominal spring constant of *k* = 0.1 Nm^−1^. The thermal noise method was used to estimate the spring constant of the cantilevers (Hutter and Bechhoefer 1993). At least 100 force‐distance curves were conducted in a 100 × 100 μm^2^ grid with a 10 μm spacing between measurement positions. The probing speed was fixed at 100 nms^−1^. For studying the systems, measurements were carried out at least three times in an open stage by depositing a drop of solution on the sample and cantilever. The media used were Milli‐Q water and NaCl (Sigma‐Aldrich) laboratory‐prepared solutions of 1, 10, and 100 mM. High‐resolution imaging was carried out with a multimode AFM (Bruker, USA) using contact mode at 295.15 K in water and NaCl solutions. The cantilevers used were MSCT silicon nitride triangular tips (Bruker, Germany) with a nominal spring constant of *k* = 0.03 Nm^−1^. The applied setpoint force was less than 1 nN. NanoScope Analysis 1.5 software was used for imaging analysis.

### 2.2 AFM Cantilever Functionalization

The cantilevers were treated in UV/ozone for 1 h prior to functionalization. Afterwards, single silica particles of a mean diameter of 9.98 ± 0.31 μm (lot: SiO_2_‐F‐SC101‐1, microParticles GmbH, Germany) were glued to cantilevers and then curated under UV light for 1 h. Following this, the SiO_2_ particle‐functionalized cantilevers were treated for 1 h in UV/ozone. The functionalized cantilevers were then placed in a desiccator chamber next to an open vessel filled with 3 mL of FOTS (1H,1H,2H,2H‐perfluorooctyltrimethoxysilane 97%, AB108485, abcr GmbH, Germany). The pressure in the chamber was reduced using a Laboport N96 pump (KNF, Germany) and the chamber was then kept sealed at 295.15 K for 19 h. UV/ozone cleaned silica‐functionalized cantilevers were used as control probes. In addition, single polystyrene (PS) particles with a mean diameter of 3 ± 0.01 μm (lot: 79166‐10ML‐F, Sigma‐Aldrich) were attached to the cantilevers as described above.

### 2.3 Substrate Functionalization

The coverslips were rinsed with ethanol and dried with N_2_. The coverslips were then oxygen plasma‐cleaned for 10 min. The cleaned coverslips were then placed in a desiccator chamber next to an open vessel filled with 3 mL of FOTS (1H,1H,2H,2H‐perfluorooctyltrimethoxysilane 97%, AB108485; abcr GmbH, Germany). The chamber was depressurized using a Laboport N96 pump (KNF group, Germany) and then sealed for 19 h at 295.15 K. Oxygen plasma‐cleaned coverslips were used as control substrates.

### 2.4 Sample Characterization by Contact Angle

Functionalized substrates were characterized using the drop sessile method. Measurements were performed at least three times with 10 μL Milli‐Q water drops at 295.15 K. The drop was deposited on the substrate using a micropipette. The same measurements were carried out on EtOH‐cleaned and N2‐dried coverslips as a control. Contact angles were determined using a Kruess EasyDrop instrument (Kruess, Hamburg, Germany).

### 2.5 Particle Characterization by Scanning Electron Microscopy (SEM)

The functionalized cantilevers with PS and FOTS particles were examined with an Apreo VS scanning electron microscope (Thermo Scientific, The Netherlands) at high vacuum using backscattered electrons (BSE) and an acceleration voltage of 1 kV.

### 2.6 Particle Characterization by Electrophoretic Light Scattering

Zeta potential measurements were performed using a Malvern Zetasizer NanoZS (Malvern Instruments, UK) equipped with a red laser (632.8 nm) and a polycarbonate closed capillary electrophoresis cell (DTS1070) with gold‐plated copper electrodes. The cell was filled with 1 mL of a freshly prepared and vortex‐dispersed sample. Measurements were carried out at least three times at 295.15 K. The silica samples (1 mL) were prepared as follows: SiO_2_ particles (5% w/v, 10 mL) were dispersed 1/10 in Milli‐Q water, NaCl 100, 10, and 1 mM. The polystyrene (PS) samples (1 mL) were prepared as follows: PS particles (10% w/v, 10 mL) were dispersed 1/100 in Milli‐Q water, NaCl 10 mM, and 1 mM.

### 2.7 AFM Data Processing and Averaging

Force quantification requires to identify the contact point (i.e., distance zero) and to correct the height of the piezo scanner to obtain the probe‐substrate separation (Figure S1). This can be performed with specialized software (i.e., JPK software) or homemade programs (Benitez et al. 2017). In our case, we carried out the analysis with JPK Data Processing SPM‐5.0.133 software (JPK Instruments AG, Germany). Thus, for the repulsive interaction between bare substrates, we distinguished the contact point at the intersection between the linear fit of the piezo height and cantilever vertical deflection, and the zero‐force line (Figure S1A). However, for hydrophobic functionalized substrates, we defined the contact point at the end of the probe jump‐in on the hydrophobically modified substrate (Figure S2A–D). Next, to simplify the analysis due to the considerable number of measured data (up to 100 curves per system), the processed force‐distance curves were averaged using the linear interpolation method with the OriginPro 2024 software (OriginLab Corporation, USA).

### 2.8 Force‐Distance Curve Simulation and Data Fitting

The DLVO theory describes the interaction forces between particles combining van der Waals (vdW) attraction and electrical double‐layer (EDL) repulsion (Israelachvili 2011). Thus, assuming low (< 50 mV) and identical surface potentials in both probe and sample (symmetric system), an expression for a system with a geometry of a spherical particle and a flat surface is described in Equation (1) (Butt et al. 2005):

(1) $$F=-\frac{A_{H}R}{6D^{2}}+\frac{4\pi \varepsilon \varepsilon_{0}\psi^{2}}{\lambda_{D}}\exp^{-\frac{D}{\lambda_{D}}}$$

where *A*_*H*_ is the Hamaker constant, *R* is the particle radius, *D* is the particle‐sample distance, *ε* and *ε*_*0*_ are the dielectric constants of solvent and vacuum, respectively, *ψ* is the surface potential and *λ*_*D*_ is the Debye length. The latter can be calculated for monovalent salt (i.e., NaCl) with Equation (2):

(2) $$\lambda_{D}=\sqrt{\frac{\varepsilon \varepsilon_{0}k_{B}T}{2ce^{2}}}$$

where *c* is the salt concentration, *k*_*B*_ is the Boltzmann's constant, *T* is the temperature, and *e* is the elementary charge.

However, DLVO theory does not account for additional interactions arising from structural effects within the intervening medium between interacting surfaces (Israelachvili and Wennerström 1996). For instance, such effects include repulsive forces observed between hydrophilic surfaces in water, commonly referred to as hydration forces (Valle‐Delgado et al. 2005). Moreover, DLVO theory does not predict the attractive interaction between hydrophobic surfaces. The origin of hydrophobic forces is particularly complex, with various mechanisms proposed. These include entropic effects attributed to water structure rearrangement, polarization effects associated with water proton hopping, local charge fluctuations, and bubble and vapor cavity bridging (Hammer et al. 2010; Wang et al. 2023). Despite their complexity, both hydration and hydrophobic interactions are typically short‐range, decaying exponentially with distance according to the following empirical relationship (Donaldson Jr. et al. 2015):

(3) $$\;F=R\;C\;\exp^{-\frac{D}{\lambda }}\;$$

In Equation (3), *C* is a pre‐exponential constant related to the interfacial tension and surface properties, while *λ* is the decay length, which also depends on the surface and solution properties. The combination of Equations (1) and (3) allowed the fitting of the experimental data as well as the curve simulation. The calculations were performed using OriginPro 2024 software (Originlab corporation, USA).

### 2.9 System Preparation and Characterization

To study the influence of salt concentration between hydrophobic surfaces, we used a C8‐fluorocarbon reagent (FOTS, see Sections 2.2, 2.3). For this purpose, FOTS properties are convenient, as they are highly hydrophobic and relatively volatile at room temperature when pressure is reduced, allowing the use of chemical vapor deposition (CVD). This technique can be used to produce smooth functionalized surfaces on silica substrates for AFM studies. An analogous hydrocarbon reagent, trimethoxyoctylsilane, has been used for similar studies (Liu et al. 2017). The CVD process of silanes, both on crystal coverslips and amorphous silica particles, involves the formation of siloxane bonds via hydrolysis and condensation reactions (Sypabekova et al. 2023). Similar hydrophilic surfaces, such as mica, can be functionalized using the same methodology.

**FIGURE 1:**
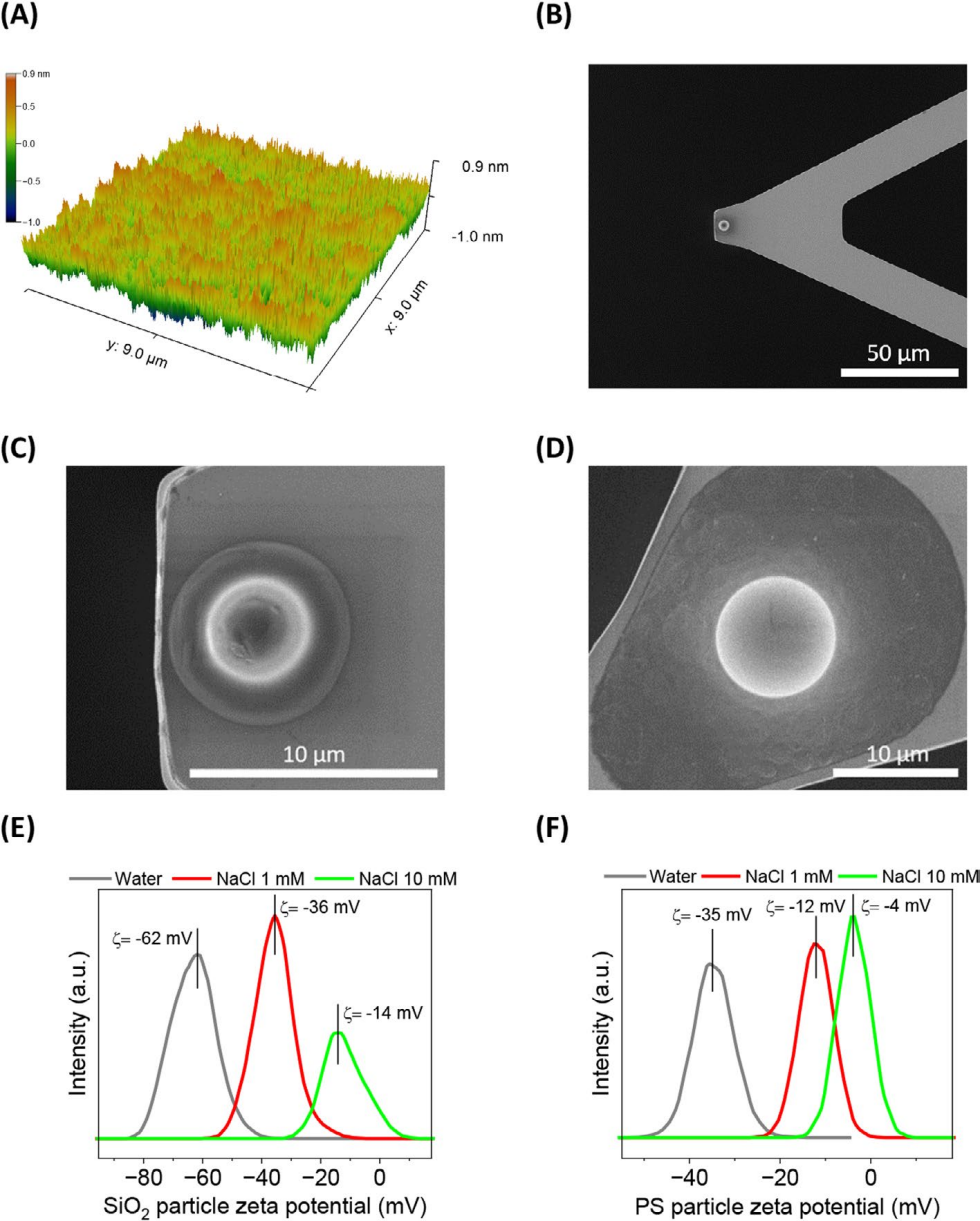
(A) Representative 3D AFM topographical image of the FOTS sample in salt solution. (B) SEM image of a Si_3_N_4_ AFM cantilever with a polystyrene particle attached. (C) SEM showing the details of the polystyrene particle. (D) Detailed SEM image of the FOTS particle attached to the Si_3_N_4_ cantilever. (E) Zeta potential distributions of silica particles in water, NaCl 1, and NaCl 10 mM. (F) Zeta potential distributions of polystyrene (PS) particles in water, NaCl 1, and NaCl 10 mM.

Hydrophobic surfaces can be defined by a water contact angle greater than 90° (Law 2014). The FOTS‐modified samples prepared by CVD exhibited a contact angle of 95° (Figure S3A). Deposition time studies revealed that the hydrophobization of the substrate is reaching a plateau after 19 h (Figure S3B). In contrast, the ethanol‐cleaned control coverslips displayed a much lower contact angle of 42° (Figure S3C) due to their hydrophilic nature. AFM imaging was also performed to analyze the topography of the samples. Therefore, Figure 1A shows a representative AFM image of a FOTS sample showing a homogeneous surface with no evidence of silane polymerization. Furthermore, the analysis showed the stability of the hydrophobic coating at different salt concentrations (Table 1) with a constant surface roughness (i.e., *R*_*q*_ from 0.22 to 0.25 nm) like the surface roughness of a bare coverslip in water: *R*_*q*_ = 0.24 ± 0.04 nm.

**TABLE 1:** Root mean square surface roughness (*R*_*q*_) and arithmetic mean surface roughness (*R*_*a*_) values obtained by AFM in NaCl solutions (1, 10, and 100 mM) for the FOTS sample.

Sample parameter	FOTS‐NaCl 1 mM	FOTS‐NaCl 10 mM	FOTS‐NaCl 100 mM
*R*_*q*_ (nm)	0.22 ± 0.03	0.23 ± 0.03	0.25 ± 0.03
*R*_*a*_ (nm)	0.18 ± 0.02	0.18 ± 0.02	0.20 ± 0.02

The particle functionalized cantilevers were then examined by scanning electron microscopy (SEM). Figure 1B shows a perspective SEM image of a PS particle correctly attached to the edge of a Si_3_N_4_ cantilever, ready for being used in probing experiments. Figure 1C exhibits a close‐up SEM image of the PS particle where its roughness and heterogeneous surface can be observed. In comparison, the SEM micrograph in Figure 1D shows a much smoother and more homogeneous spherical FOTS particle.

To characterize the SiO_2_ substrate further, a suspension of SiO_2_ particles was studied by electrophoretic light scattering to obtain zeta potential values. This information is useful for understanding the stability of a colloidal system over a range of salt concentrations. A negative zeta potential was obtained in water (−62 mV), which decreased with increasing salt concentration (−14 mV, NaCl 10 mM, Figure 1E). However, no zeta potential distribution was observed for the SiO_2_ particle dispersion in NaCl 100 mM (point of zero net charge at the shear plane). The zeta potential of the hydrophobic particles was then investigated. Figure 1F shows that the negative zeta potential of polystyrene (PS) particles in water (−35 mV) also decreased with increasing salt concentration (−4 mV, NaCl 10 mM).

## 3 Results and Discussion

### 3.1 Symmetric Systems

#### 3.1.1 Experimental Results

In colloidal systems, such as silica and polystyrene particles in water, the particles are charged and the presence of salt in the solution can screen these charges, thereby affecting the repulsive forces between the particles. Adding salt to the system reduces the electrostatic repulsion, which can lead to particle aggregation (note that although this repulsion is called electrostatic, it is actually entropy driven, as described in (Israelachvili 2011)). Understanding and controlling these interactions is essential for achieving colloidal stability. In addition, the study of colloids covered with hydrophobic coatings can help to elucidate how emulsions, surfactant‐stabilized, and bio self‐assembled systems behave in liquid media.

We conducted AFM force‐distance experiments on a symmetrical system comprising a silica particle and a coverslip, both coated with a hydrophobic reagent (FOTS). Measurements were performed at 295.15 K in NaCl solutions of different concentrations (1, 10, and 100 mM). Analogous experiments were performed for the hydrophilic SiO_2_ particle–coverslip system.

**FIGURE 2:**
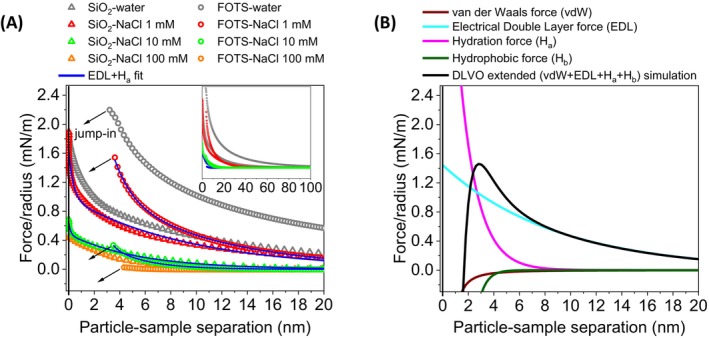
(A) Radius normalized average force‐distance approaching curves between SiO_2_ particles and coverslip sample (hollow triangle) and FOTS‐coated SiO_2_ particles and FOTS‐coated coverslip sample (hollow dot) at a probing speed of 100 nm s^−1^ in water, NaCl 1, NaCl 10, and NaCl 100 mM. The blue solid line represents electrical double‐layer (EDL) plus hydration (H_a_) fitting. (B) DLVO extended (black solid line) radius normalized simulated force‐distance curve for the FOTS symmetric system at NaCl 1 mM. Magenta, cyan, brown and dark green lines represent the contributions of hydration (H_a_), electrical double‐layer (EDL), van der Waals (vdW) and hydrophobic (H_b_) interactions, respectively.

As shown in Figure 2A, long‐range repulsive forces dominated the interaction at large separations in all the systems, gradually attenuating with increasing salt concentration. For example, in the FOTS‐FOTS system, the range of repulsive forces decreased from up to 100 nm in water to less than 10 nm at NaCl 100 mM. However, a fixed short‐range jump to contact was observed on average at a distance of about 3–4 nm, independent of the salt concentration. In contrast, for the SiO_2_‐SiO_2_ system, repulsion persisted during the entire approach and steepened at very close separations, consistent with hydration repulsion.

The long‐range repulsion arises from double‐layer forces due to ionized silanol groups on the surface. Nevertheless, the surface charge can remain after hydrophobization due to unreacted hydroxyl groups (Liu et al. 2017). The observed short‐range attraction was identified as hydrophobic force. Short‐range hydrophobic jump‐in (< 10 nm) has been described in other works with similar systems and conditions (Yoon et al. 1997; Liu et al. 2017; Kage et al. 2023). The gradient of the attractive interaction‐van der Waals plus hydrophobic‐is strong enough to overcome the spring constant of the cantilever ($\frac{\mathrm{dF}}{\mathrm{dz}}>k$), causing the probe to jump into contact with the sample. This makes it difficult to discriminate between van der Waals and hydrophobic forces, even for stiffer cantilevers (e.g., 20 N m^−1^). However, when probing with nanometric tips, exponentially decaying data has been obtained instead of jump‐in (Stock et al. 2015; Xie et al. 2016).

To obtain the constants associated with the electrostatic interactions (i.e., the surface potential and the Debye length), we fitted the experimental data to the electrical double‐layer (EDL) model (Equation (1), 2nd term). Table S1 lists the parameters obtained from the calculations. Correspondingly, Figure S4 shows that the EDL fits were rather poor for the SiO_2_–SiO_2_ system due to steep short‐range repulsion attributed to non‐DLVO forces (i.e., hydration forces). These forces, caused by disruption of the water structure near hydrophilic surfaces (e.g., hydration layers) have been fitted using exponential decay models (Yoon et al. 1997; Considine and Drummond 2001; Butt et al. 2005; Donaldson Jr. et al. 2015).

To improve the fit of the data, we included a hydration force term, H_a_ (Equation (3)). In this direction, the results (Figure 2A, Table 2) show how the inclusion of the hydration force significantly improves the fit of the experimental data, yielding a more consistent model. The extended DLVO equation provided fitted EDL parameters closer to the calculated Debye lengths with Equation (2) (*λ*_*D*_ = 9.5 nm in NaCl 1 mM and *λ*_*D*_ = 3.02 nm in NaCl 10 mM at 295.15 K), and measured zeta potentials. At low ionic strengths, where the electrical double‐layer is thin, and both the particle surface and the slip plane are close, the surface potential and the zeta potential should have similar values.

**TABLE 2:** Parameters used in van der Waals (vdW), hydration force (H_a_), electrical double‐layer (EDL) and hydrophobic force (H_b_) calculations.

System parameter	FOTS‐FOTS (NaCl 1 mM)	FOTS‐FOTS (NaCl 10 mM)	SiO_2_‐SiO_2_ (NaCl 1 mM)	SiO_2_‐SiO_2_ (NaCl 10 mM)
Hamaker constant (A_H_)/J ×10^−20^	0.40	0.40	0.85	0.85
Hydrophobic force constant (C_b_)/mN m^−1^	−50	−50	—	—
Hydrophobic force decay length (λ_b_)/nm	0.6	0.6	—	—
Debye length (λ_D_)/nm	8.9	2.9	9.5	5.5
Surface potential (ψ)/mV	−38	−17	−32	−17
Hydration force constant (C_a_)/mN m^−1^	7.1	52	0.64	0.17
Hydration force decay length (λ_a_)/nm	1.4	0.072	0.36	0.14

The hydration force parameters for the SiO_2_‐SiO_2_ system showed a decay length of < 1 nm and did not vary significantly with salt concentration (see Table 2), since silica does not possess ion exchange centers, and the repulsive behavior is more steric (Donaldson Jr. et al. 2015). Interestingly, although the hydration of a hydrophobic surface seems counterintuitive, a steep short‐range repulsive interaction was also observed for the FOTS‐FOTS system. This repulsive force could result from the combined effects of water depletion forces and gradients of confined water molecules or hydrated ions between the vicinal ionized silanols and grafted fluorocarbon chains.

#### 3.1.2 Curve Simulation

DLVO extended curves were simulated to discuss how the contributions of both van der Waals (vdW) and hydrophobic forces (H_b_) might have affected the interactions in the studied systems. Therefore, Equation (1), which includes vdW and EDL, was used in combination with Equation (3), which includes the hydration force (H_a_) and the hydrophobic force (H_b_). The Hamaker constants (A_H_) were selected from tabulated sources for both silica (Hough and White 1980) and the hydrophobic carbon chain (Evans and Wennerström 1999) in water.

As with the hydrophobic force parameters, the interfacial tension between the hydrophobic phase and water was selected as a constant (*C*_*b*_ = −50 mN m^−1^) and the decay length was conveniently chosen (*λ*_*b*_ = 0.6 nm) as it has been reported to vary between 0.3 and 1 nm (Donaldson Jr. et al. 2015). Since the jump‐in remains constant in the experimental data, we assume a similar hydrophobic strength constant and force decay for both NaCl 1 and NaCl 10 mM. All other parameters were taken from the fits to the experimental data.

Figure 2B shows the extended DLVO simulation along with the contribution of each individual term for the FOTS‐FOTS system in NaCl 1 mM. This visual composition of the forces can help to understand the role of vdW and H_b_ in the overall interaction. In particular, both contributions are attractive forces and operate in a similar range and within approximately the same separation distance as the observed experimental jump‐in (3–4 nm). This suggests that H_b_ may have a similar nature to vdW, but with a stronger polarizing effect. Accordingly, it has been found that long‐range dipolar correlation and coupling of water at the hydrophobic surfaces can generate attractive interaction (Despa and Berry 2007; Kanth et al. 2010).

Additionally, the simulated DLVO curves (Figure S5) reflect the weak contribution of the vdW attractive interactions to the SiO_2_‐SiO_2_ experimental data, which have also been reported for silica‐silica and gold–gold surfaces (Considine and Drummond 2001; Wang and Yoon 2008). Complementing the empirical data, the simulations show how the vdW attraction could theoretically overcome the repulsive hydration forces, since both interactions occur in a similar range. Nevertheless, the contribution of the repulsive hydration forces could stabilize the system. Moreover, it has been proposed that the attenuation of the vdW attractive forces may be due to the imperfect structure of the interacting surfaces (e.g., nanometric asperities) (Considine and Drummond 2001).

### 3.2 Asymmetric Systems

The previous section showed that in a symmetric hydrophobic system (FOTS‐FOTS), the substrate properties in media (e.g., silica surface potential) govern the long‐range interactions despite the hydrophobic coating. In this section, we investigate asymmetric systems. Instead of using a hydrophobized silica probe, we have directly used a bare hydrophobic particle (polystyrene, PS) (Xu et al. 2007).

**FIGURE 3:**
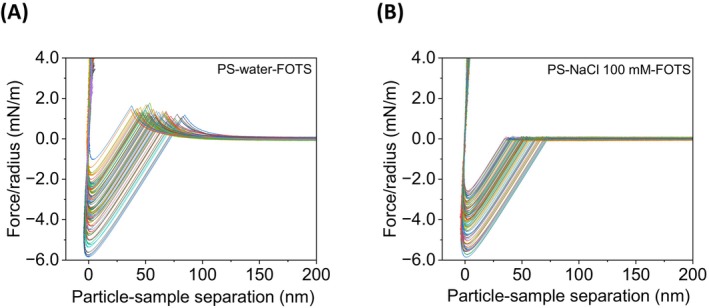
Radius normalized force‐distance approaching curves between PS particle and FOTS‐coated coverslip sample at a probing speed of 100 nm s^−1^ in water (A) and NaCl 100 mM (B). Note how the repulsive region of (A) is strongly reduced in the presence of NaCl (B). The depth of the minimum is similar in both systems.

As can be seen in Figures 3 and S6, long‐range repulsive forces occur between the PS particle and the substrate in both the bare and FOTS‐hydrophobized coverslips. In the case of the PS‐SiO_2_ system, the repulsion in water was regular, with an average range of about 100 nm (inset, Figure S6). However, for the PS‐FOTS system in water (Figure 3A), the repulsive interaction range showed a large variation due to the presence of irregular hydrophobic attraction. Furthermore, Figure 3B shows how this long‐range repulsion is strongly attenuated in the presence of NaCl at a high concentration (100 mM), in agreement with the phenomena observed in the symmetric systems. Therefore, the long‐range repulsive interaction between both PS‐SiO_2_ and PS‐FOTS asymmetric systems is ascribed to the electrical double‐layer forces between the two negatively charged substrates.

The attractive interaction between PS and FOTS shows a significant variation in the jump‐in to contact distance values in water (38–85 nm) and in NaCl 100 mM (35–73 nm), which correlates with their respective net jump‐in forces fitting a slope close to the nominal spring constant of the cantilever (see Figure S7). In addition, this attractive force was absent in the PS‐SiO_2_ system, representing a hydrophobic‐hydrophilic system (Figure S6), where the repulsive interaction completely dominates, as reported for a PS‐silica system in water (Thormann et al. 2008) and a PS‐mica system in salt solution (Faghihnejad and Zeng 2013). Subsequently, the irregular interactions in the asymmetric hydrophobic system (PS‐FOTS) could be caused by the roughness of the PS particle surface. The heterogeneous hydrophobic surface could interact in a different range depending on the probed region of the hydrophobic FOTS sample surface. For example, these long‐range and heterogeneous attractive interactions between hydrophobic surfaces have been reported as to be generated by the formation of vapor or gas cavities (e.g., micro and nanobubble bridging) on hydrophobic rough surfaces (Nalaskowski et al. 1999; Nguyen et al. 2003; Azadi et al. 2015).

Cavitation‐induced capillary bridging can be increased after repeated approach‐retract cycles between the surfaces, leading to changes in the jump‐in distance (Azadi et al. 2015). Furthermore, the variation in jump‐in distance could also be influenced by the difference in gas solubility from water to salt solution (Azadi et al. 2015). Nevertheless, despite the still inconclusive origin of the hydrophobic attraction, we observed that the distance at which jump‐in occurs seems to be related to the morphology of the hydrophobic substrate (i.e., surface roughness).

## 4 Conclusions

In this primer, we investigated the influence of salt on colloidal forces between hydrophobic and hydrophilic surfaces using atomic force microscopy (AFM). Force‐distance experiments on symmetric hydrophobic (FOTS‐FOTS) and hydrophilic (SiO_2_‐SiO_2_) systems revealed different interaction regimes. Long‐range electrostatic repulsion dominated at large separations in all systems, attenuated by increasing salt concentration, followed by a short‐range steep hydration repulsion. Additionally, the FOTS‐FOTS system exhibited a short‐range hydrophobic attraction characterized by a fixed jump‐in at ca. 4 nm, independent of the salt concentration.

In asymmetric systems, such as polystyrene (PS) particles interacting with either FOTS or silica, the long‐range repulsion was also attenuated by salt concentration, while the hydrophobic attraction in PS‐FOTS interactions displayed irregular and longer jump‐in distances (35–85 nm). The difference in jump‐in distance observed between FOTS‐FOTS and PS‐FOTS systems in force‐distance experiments, combined with topography analysis, suggests that the jump‐in distance may be influenced by surface roughness.

Fitting the experimental data with DLVO theory demonstrated the need to use an extended model even for “simple” substrate‐modified systems. The combination of van der Waals, electric double‐layer, hydration, and hydrophobic forces allowed the calculation of parameters such as surface potential and Debye length. Furthermore, an extended DLVO curve simulation was performed to gain insight into the interaction mechanisms.

As a future scope, new experiments will be carried out in hydrophobic systems to investigate the influence of water structure stabilization and disruption using nonionic kosmotropic agents (e.g., glucose) and ionic chaotropic agents (e.g., guanidinium chloride), respectively. In addition, regression curves can provide more information on hydrophobic forces through adhesion studies combined with contact angle at different solvent polarity, a work that is ongoing.

## Author Contributions

**Luis N. Ponce‐Gonzalez:** investigation, writing – original draft, formal analysis, data curation, methodology, conceptualization. **Wisnu Arfian A. Sudjarwo:** investigation, writing – review and editing, methodology, data curation. **José L. Toca‐Herrera:** conceptualization, funding acquisition, writing – original draft, writing – review and editing, validation, formal analysis, project administration, supervision, resources.

## Supporting information


Data S1.

## Data Availability

The data that support the findings of this study are available on request from the corresponding author. The data are not publicly available due to privacy or ethical restrictions.
