# Supplementary material for: Measuring Colloidal Forces With Atomic Force Microscopy 1: Salt Influence on Hydrophobic and Hydrophilic Interactions
Source: Microsc Res Tech. 2025 Feb 22;88(6):1626–34. doi: 10.1002/jemt.24832 (PMC12076107; doi:10.1002/jemt.24832)
Supplement: Supplementary file 1 — Data S1. [file JEMT-88-1626-s001.docx]

**Supporting Information**

**Measuring colloidal forces with AFM 1: salt influence on hydrophobic and hydrophilic interactions**

Luis N. Ponce-Gonzalez,^1^ Wisnu Arfian A. Sudjarwo^1,2^ and José L. Toca-Herrera*^1^

^1^Institut für Biophysik, Department für Bionanowissenschaften, Universität für Bodenkultur Wien, Muthgasse 11, 1190 Vienna, Austria.

^2^Research Center for Polymer Technology, National Research and Innovation Agency, Republic of Indonesia (BRIN), Serpong, Tangerang Selatan 15314 Indonesia.

Email: [jose.toca-herrera@boku.ac.at](mailto:jose.toca-herrera@boku.ac.at)

Contents

[**Atomic force microscopy (AFM) data processing** 1](#_Toc188955687)

[**Raw force-distance curves of hydrophobic symmetric system** 2](#_Toc188955688)

[**Contact angle measurements** 3](#_Toc188955689)

[**Electrical double-layer (EDL) fitting (including Table S1)** 4](#_Toc188955690)

[**Force-distance curve simulation** 5](#_Toc188955691)

[**Average curves of polystyrene-SiO_2_ system** 6](#_Toc188955692)

[**Jump-in distance-force correlation** 7](#_Toc188955693)

# **Atomic force microscopy (AFM) data processing**

| **(A)**  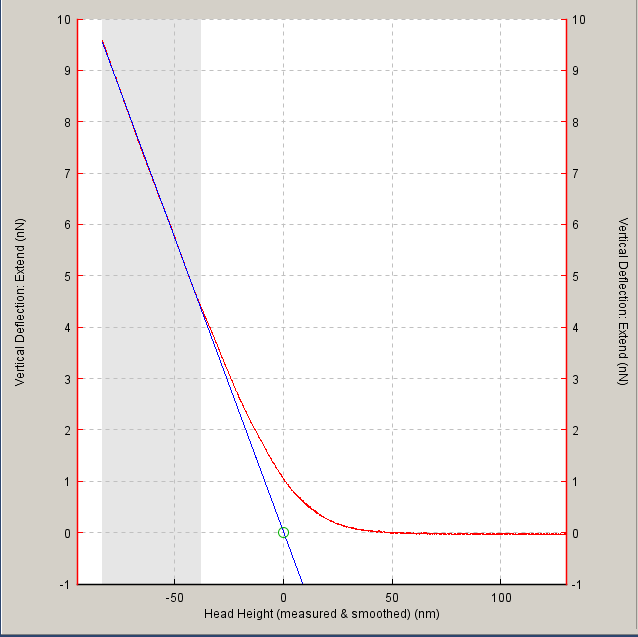 | **(B)**  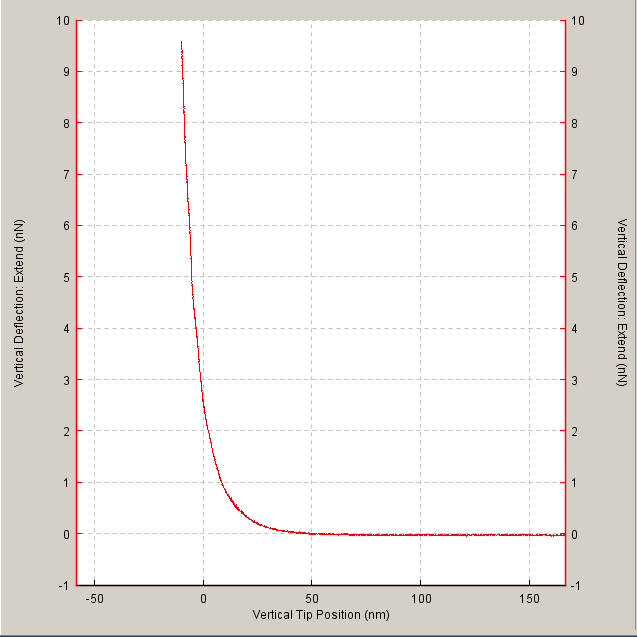 |
| --- | --- |

**Figure S1.** Example of the extended segment of a force-distance curve between bare surfaces in liquid media processed with JPK software. (A) Illustrates how the contact point (x=0) is determined at the intersection of the linear fit of the piezo height-vertical deflection and the zero-force line. (B) Depicts the probe-substrate distance after adjusting for the height of the piezo scanner.

# **Raw force-distance curves of hydrophobic symmetric system**

| **(A)**  **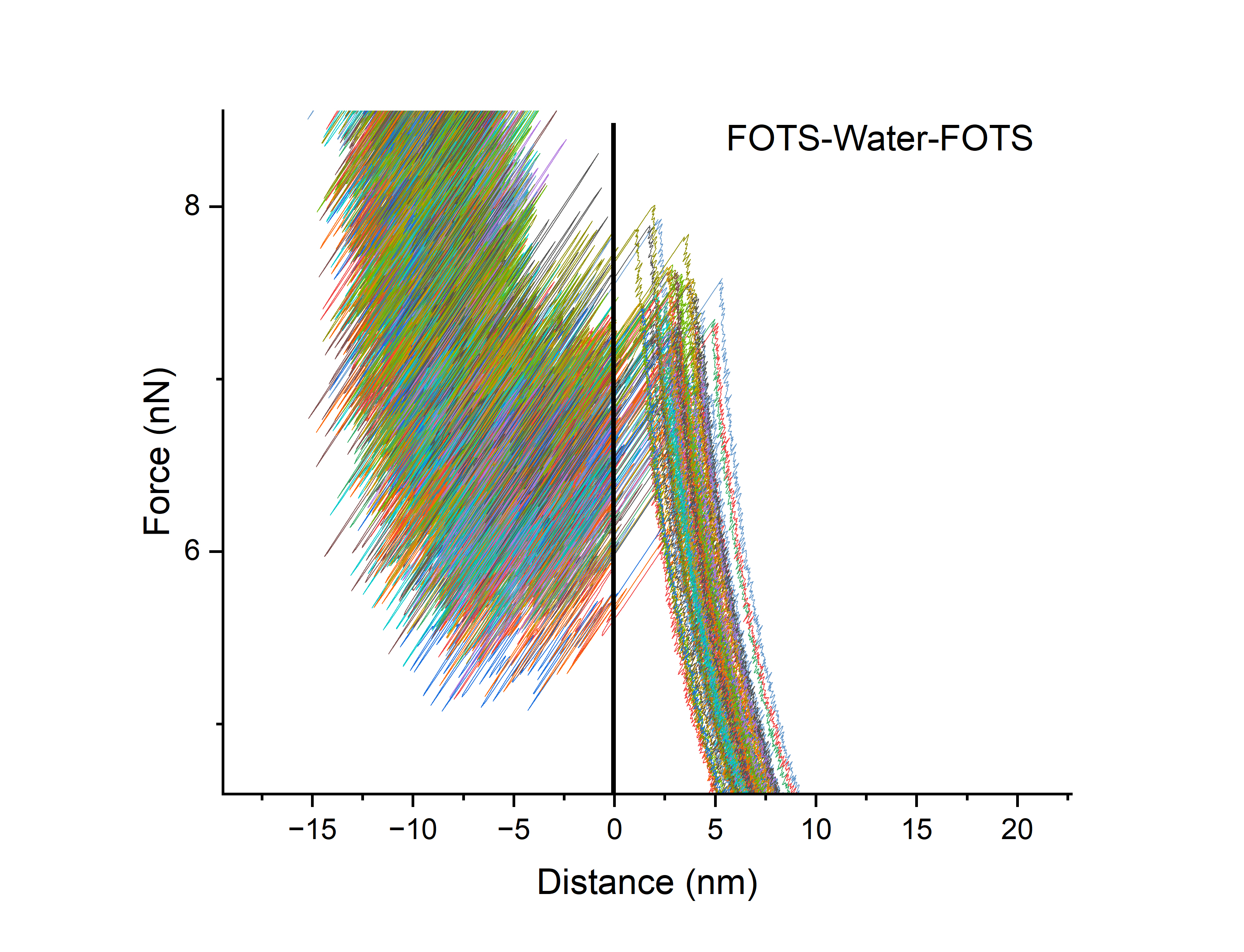** | **(B)**  **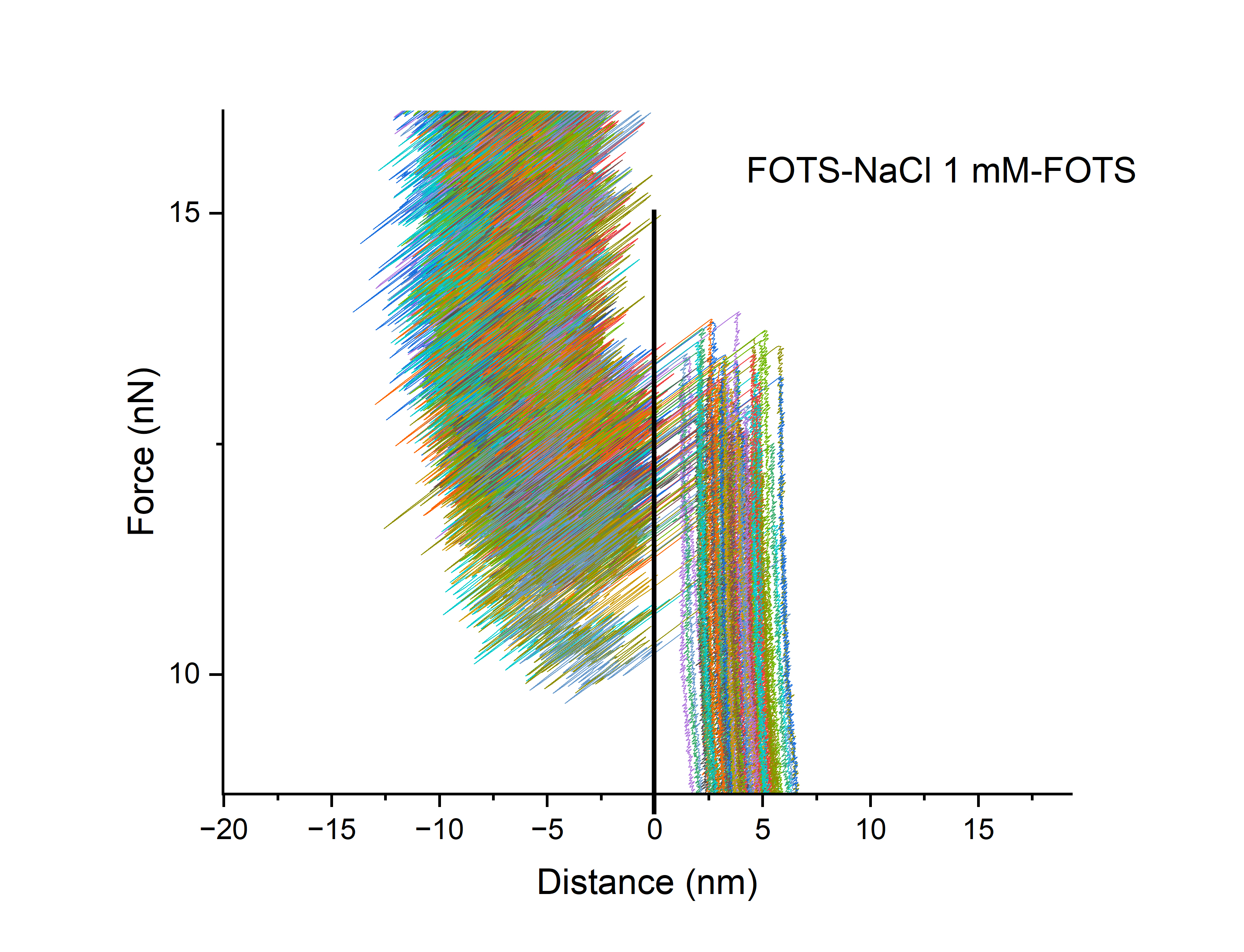** |
| --- | --- |
| **(C)**  **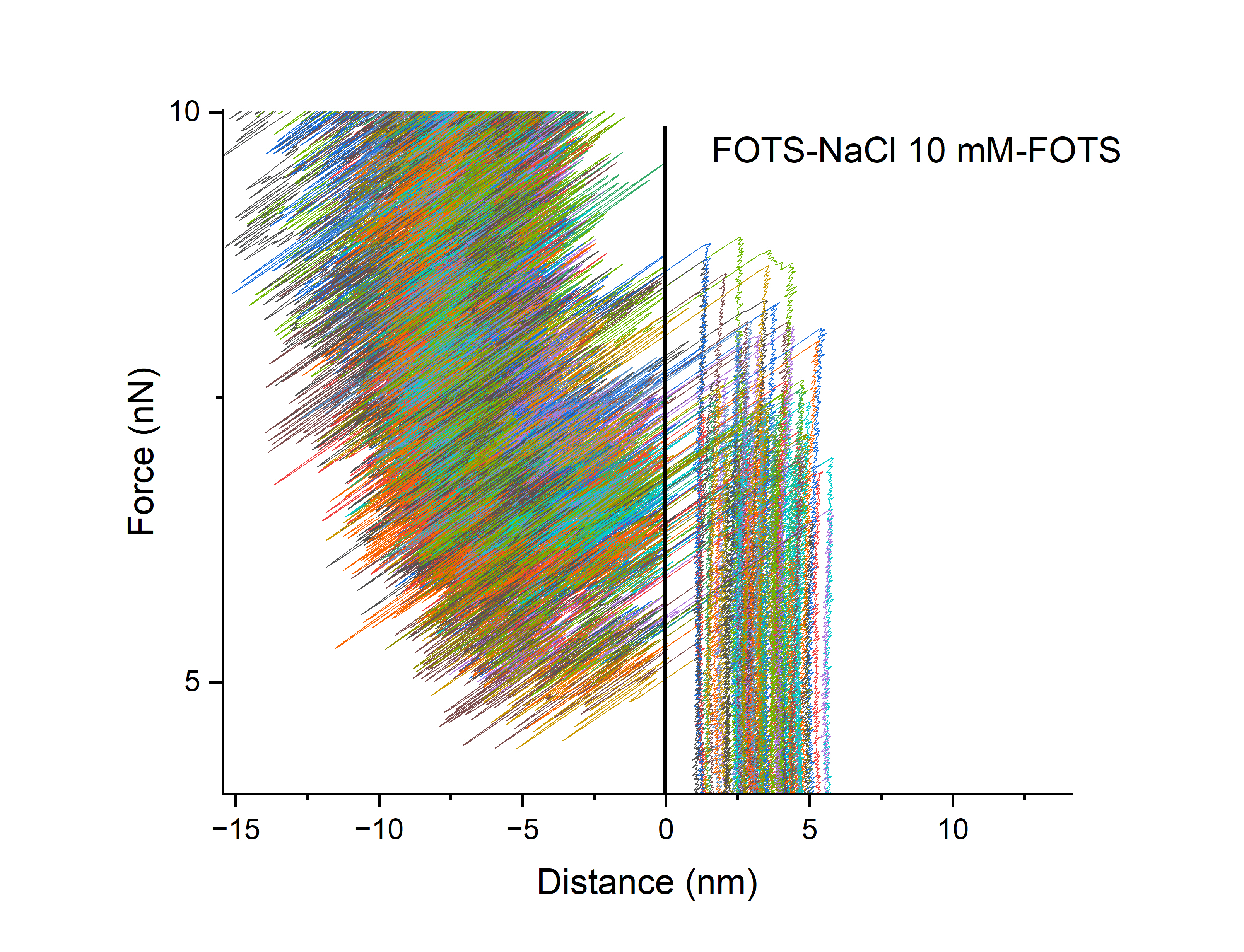** | **(D)**  **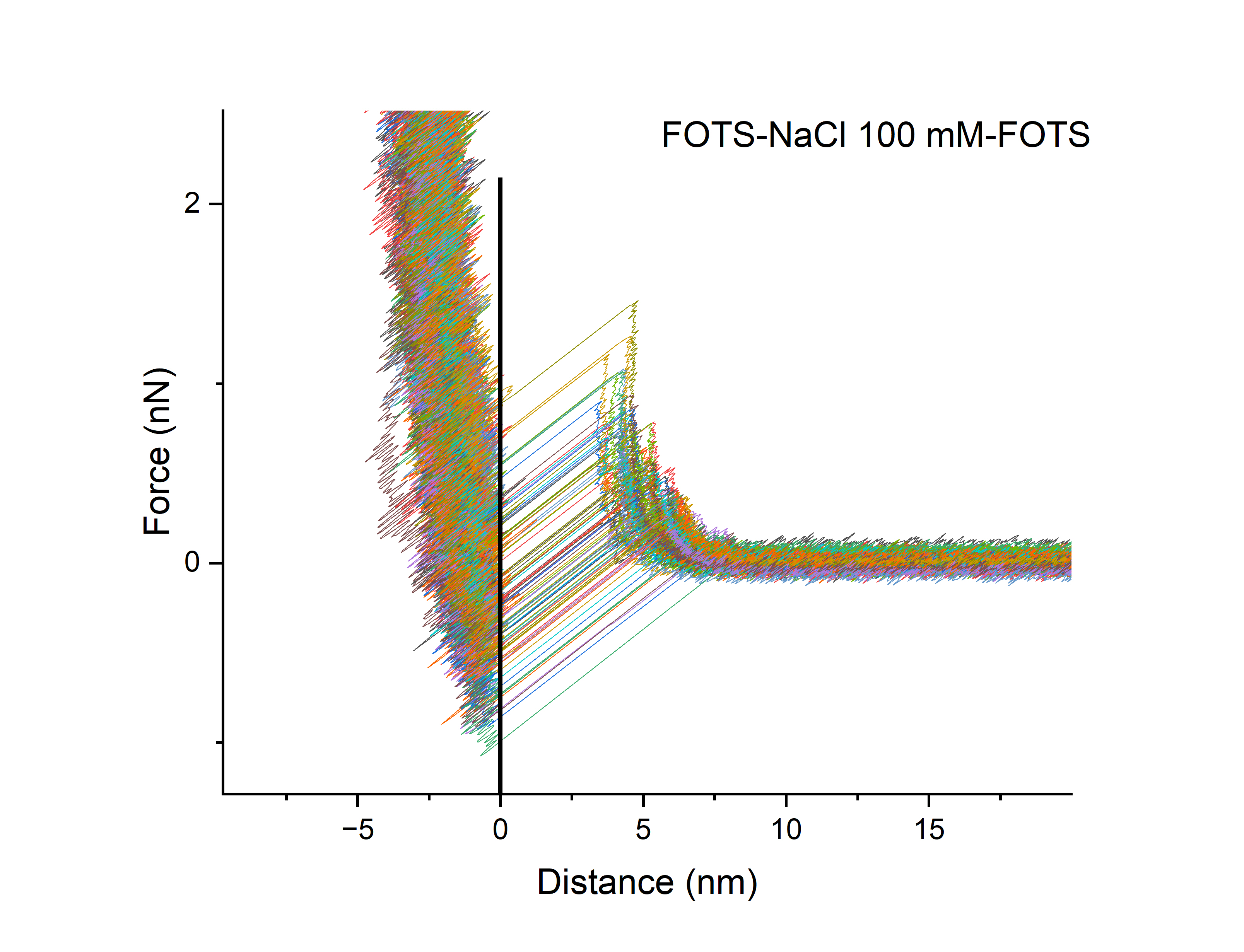** |

**Figure S2.** Representative approach force-distance curves between a FOTS-coated coverslip and a FOTS-coated silica particle at a probing speed of 100 nm s^-1^ in water (A), NaCl 1 mM (B), NaCl 10 mM (C), and NaCl 100 mM (D). The vertical black line indicates the contact point at the end of the jump-in. The temperature of the experiment was 295.15 K.

# **Contact angle measurements**

| **(A)**  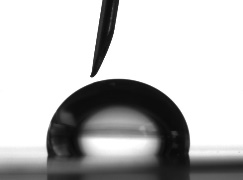 | **(B)**   |
| --- | --- |
| **(C)**  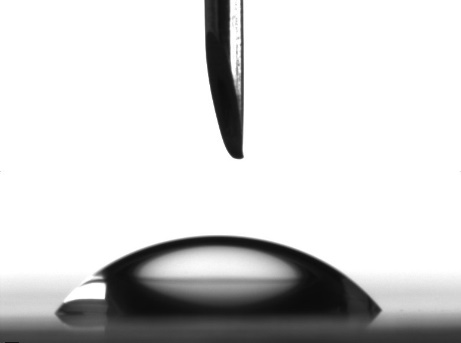 |  |

**Figure S3.** Image of a 10 µL water drop on a FOTS coated coverslip sample after 19 hours of FOTS chemical vapour deposition (A). Time course studies of FOTS chemical vapour deposition (B). Image of a 10 µL water drop on a bare coverslip sample cleaned with ethanol (C). The temperature of the experiments was 295.15 K.

# **Electrical double-layer (EDL) fitting (including Table S1)**

**Figure S4.** Radius normalized average force-distance approaching curves between a SiO_2_ particle and a coverslip sample (hollow triangle) and between a FOTS-coated SiO_2_ particle and a FOTS-coated coverslip sample (hollow dot) at a probing speed of 100 nm s^-1^ in NaCl 1 mM (red) and NaCl 10 mM (green). The magenta solid line represents electrical double-layer (EDL) fit of the experimental data. The temperature of the experiment was 295.15 K.

**Table S1.** Parameters derived from the electrical double-layer (EDL) fitting of the experimental systems.

| System Parameter | FOTS-FOTS  (NaCl 1 mM) | FOTS-FOTS  (NaCl 10 mM) | SiO_2_-SiO_2_  (NaCl 1 mM) | SiO_2_-SiO_2_  (NaCl 10 mM) |
| --- | --- | --- | --- | --- |
| Debye length/nm  (EDL fitting) | 6.9 | 2.6 | 4.9 | 4.6 |
| Surface potential/mV (EDL fitting) | -41 | -19 | -28 | -17 |

# **Force-distance curve simulation**

**Figure S5.** DLVO extended radius normalized simulated force-distance curve for the FOTS symmetric system at NaCl 10 mM (black solid line), and for the SiO_2_ symmetric system at NaCl 1 mM (black dashed line) and 10 mM (black dotted line). The temperature of the experiment was 295.15 K.

## **Average curves of polystyrene-SiO_2_ system**

**Figure S6.** Average approaching radius normalized force-distance curves between a coverslip sample and a polystyrene (PS) particle at a probing speed of 100 nm s^-1^ in water (grey), NaCl 1 mM (red), NaCl 10 mM (green), and NaCl 100 mM (blue). The temperature of the experiment was 295.15 K.

# **Jump-in distance-force correlation**

**Figure S7.** Linear fit showing the relationship between the jump-in force and the jump-in distance for the system of a polystyrene (PS) particle versus a FOTS-coated coverslip sample in water at a probing speed of 100 nm/s. The temperature was 295.15 K, and the regression coefficient of the fit was 0.99.
